# Using Twitter to Examine Web-Based Patient Experience Sentiments in the United States: Longitudinal Study

**DOI:** 10.2196/10043

**Published:** 2018-10-12

**Authors:** Kara C Sewalk, Gaurav Tuli, Yulin Hswen, John S Brownstein, Jared B Hawkins

**Affiliations:** 1 Computational Health Informatics Program Boston Children's Hospital Boston, MA United States; 2 Department of Social and Behavioral Sciences Harvard TH Chan School of Public Health Boston, MA United States; 3 Department of Pediatrics Harvard Medical School Boston, MA United States; 4 Department of Biomedical Informatics Harvard Medical School Boston, MA United States

**Keywords:** health care, social media, patient experience

## Abstract

**Background:**

There are documented differences in access to health care across the United States. Previous research indicates that Web-based data regarding patient experiences and opinions of health care are available from Twitter. Sentiment analyses of Twitter data can be used to examine differences in patient views of health care across the United States.

**Objective:**

The objective of our study was to provide a characterization of patient experience sentiments across the United States on Twitter over a 4-year period.

**Methods:**

Using data from Twitter, we developed a set of 4 software components to automatically label and examine a database of tweets discussing patient experience. The set includes a classifier to determine patient experience tweets, a geolocation inference engine for social data, a modified sentiment classifier, and an engine to determine if the tweet is from a metropolitan or nonmetropolitan area in the United States. Using the information retrieved, we conducted spatial and temporal examinations of tweet sentiments at national and regional levels. We examined trends in the time of the day and that of the week when tweets were posted. Statistical analyses were conducted to determine if any differences existed between the discussions of patient experience in metropolitan and nonmetropolitan areas.

**Results:**

We collected 27.3 million tweets between February 1, 2013 and February 28, 2017, using a set of patient experience-related keywords; the classifier was able to identify 2,759,257 tweets labeled as patient experience. We identified the approximate location of 31.76% (876,384/2,759,257) patient experience tweets using a geolocation classifier to conduct spatial analyses. At the national level, we observed 27.83% (243,903/876,384) positive patient experience tweets, 36.22% (317,445/876,384) neutral patient experience tweets, and 35.95% (315,036/876,384) negative patient experience tweets. There were slight differences in tweet sentiments across all regions of the United States during the 4-year study period. We found the average sentiment polarity shifted toward less negative over the study period across all the regions of the United States. We observed the sentiment of tweets to have a lower negative fraction during daytime hours, whereas the sentiment of tweets posted between 8 pm and 10 am had a higher negative fraction. Nationally, sentiment scores for tweets in metropolitan areas were found to be more extremely negative and mildly positive compared with tweets in nonmetropolitan areas. This result is statistically significant (*P*<.001). Tweets with extremely negative sentiments had a medium effect size (*d*=0.34) at the national level.

**Conclusions:**

This study presents methodologies for a deeper understanding of Web-based discussion related to patient experience across space and time and demonstrates how Twitter can provide a unique and unsolicited perspective from users on the health care they receive in the United States.

## Introduction

In the past decade, we have observed a shift in the United States health care system to emphasize a patient-centered approach to care [1]. Standardized practices to qualitatively assess the care patients receive at hospitals have been developed, such as the Hospital Consumer Assessment of Healthcare Providers and Systems survey [2]. Many benefits to patient-centered health care facilities have been identified, including reduced length of stay, lower costs per case, decreased adverse events, and even reduced operating costs [1]. Studies have even found that better reported patient care experiences are associated with better clinical outcomes, improved safety within hospitals, and less frequent use of health care [3,4].

Traditional assessments have also documented differences in access to health care [5]. Research has shown that access to health care varies based on where a patient lives [6,7,8]. Patient care is often dependent upon the policies of the state a patient lives in, distance to the nearest health care facilities, and insurance coverage, which varies across the United States. Population size can impact many of these factors. It has been shown that individuals in large metropolitan cities tend to have better access and quality of care compared with smaller, more rural communities [6].

However, commonly used assessments of patient care, such as surveys or focus groups, have limitations that include social desirability bias, smaller audiences, and restrictions on what questions and topics patients are asked about [9,10]. The Pew Research Center reported that 87% of Americans who have seen a health care provider report positive feedback on their experience. However, 39% of US adults believe that US health care is below average [11,12].

With an increasing demand for transparency in health care, social media has shifted to become a platform for patient engagement and empowerment. Currently, there are 69 million monthly active Twitter users in the United States [13], highlighting the overwhelming use and potential for rich information to be extracted from the social networking site. Information on social media could be valuable to complement evaluations of patient care because Web-based posts provide an unsolicited, free-text perspective from users on the care they receive. There are limited studies which provide in-depth examinations of care across the United States and few, if any, that are reflections of social media discussions.

Previous research has shown that Twitter can be used as a supplemental data stream for measuring the patient-perceived quality of care in US hospitals by comparing patient sentiments about hospitals with established quality measures and traditional hospital-based feedback reports [14]. This indicates that Web-based data about patient experience and hospital care that is valuable to explore further are available from Twitter. Additionally, such research has shown that a range of topics can be identified and understood from these tweets [14,15]. Novel approaches can be used to further describe differences in hospital performances [16]. This includes sentiment analysis, a process that examines the content of free-short message service text messages and determines a score rating on a scale of positive to negative [17]. Sentiment analyses have been shown useful in describing patient opinions on hospital care that are comparable with results from more traditional survey methods [18]. An evaluation of research using sentiment analyses for health care-related tweets identified a need for improved methods of understanding sentiment data in a health care setting [17]. Previous examinations have also shown that social media research has explored specific public health topics and target populations, but there lacks a comprehensive study that fully examines a communication tool for a larger scope to evaluate population health needs [19].

To examine sentiments of health care in the United States online, we captured tweets discussing patient experiences not restricted by the level or type of health service provided. This dataset is the first of its kind that explores carefully curated data from the Twitter platform related to patient experience, which includes, but is not limited to, interactions at hospitals, urgent care facilities, primary and specialty care offices, and related health care facilities. Using this rich dataset, we aim to provide a spatial and temporal characterization of the sentiment of health care discussions on Twitter and determine if there are differences in the sentiment of health care discussions between metropolitan and nonmetropolitan areas in the United States using Twitter as a real-time supplementary data stream. Insight on patient experience discussions online can help inform health care facilities, key stakeholders and future research practices for examining patient feedback using Web-based data.

## Methods

### Patient Experience Classifier

This study utilizes data from the social media platform Twitter to investigate the experiences of patients at hospitals, urgent care facilities, primary and specialty care offices, and other related health care facilities. We used a combination of keywords to gather publicly available patient experience-related tweets through Gnip, the Twitter-owned data broker. Gnip is a paid licensing software service for Twitter data. All data collected in this study were publicly posted on Twitter; therefore, per the privacy policy of Twitter [20], users elect to have this information available to the general public for consumption. A set of keywords and rules were meticulously chosen to retrieve tweets potentially discussing experience related to the following areas: medical facilities and staff, medical procedures, hospital visits and stays, medications, hospital bills and insurance, care condition, and pain. The keywords were divided into the classes to correctly form the rules. A list of classes along with the corresponding set of keywords and example rules are shown in Multimedia Appendix 1; for example, care condition keywords include monitor, heal, recover, care, cure, dying, dead, sicker, sick, ill, illness, and condition. The keywords retrieved 27,309,724 unique tweets (45.3 million when including the retweets) posted between February 1, 2013 and February 28, 2017. The retweets were not considered in the study.

We developed a set of software components to auto label and examine the patient experience Twitter dataset. The set includes a classifier to determine patient experience tweets, a geolocation inference engine for social data, a modified version of a sentiment classifier from the literature, and an engine to determine if the tweet is from a metropolitan or nonmetropolitan area. These components were built for appropriately handling health care experience social data.

For the purpose of this study, we identified the tweets captured that were relevant to the patient experience. A relevant tweet included discussions about care received in a hospital, urgent care, or any other health institution—either by the person themselves, a friend, or relative. We aimed to capture tweets that discussed any exposure to health care.

We built a supervised machine classifier for identifying relevant patient experience tweets. A 2-step curation approach was adopted to create a training dataset for the classifier. We determined that tweets containing a Web page link (also known as URL) are 18 times more likely to be irrelevant. Two randomly selected sets of 5000 tweets, one with and the other without URLs, were hand curated using Amazon Mechanical Turk (MTurk) for this examination. The set with URLs contained only 56 of 4599 agreed upon relevant tweets (1.22%) compared with 760 of 3439 agreed upon relevant tweets (22.10%) in the set without URLs. Therefore, we decided to only consider tweets without URLs for this study. We curated 15,000 additional tweets without URLs on MTurk. In total, the manual MTurk curation gave us 3708 relevant and 9810 irrelevant patient experience tweets for which at least two of the MTurk curators were in agreement. There was an agreement on a total of 13,885 of 20,000 tweets without URLs (69.43%) between the MTurk curators. All MTurk curators selected were identified as master’s-level workers, having been monitored and verified by Amazon as high performing and demonstrating excellence in their curation tasks [21]. All MTurk curators were restricted to only curate each tweet once. Example curation instructions for the MTurk curators are presented in Multimedia Appendix 2. A few examples of manually curated tweets are shown in Table 1. The tweets provided in this table are fictitious examples to preserve user identity and privacy, a technique that has been recommended in previous research to address the ethical concerns of disseminating Twitter data [22].

We developed a support vector machine-based supervised machine learning classifier using this training set to filter relevant tweets from irrelevant ones. The classifier was built using various textual features and was iteratively evaluated using the 10-fold cross validation over 90% training and 10% test sets. Each training tweet was tokenized using the Natural Language Toolkit TweetTokenizer. Stop words and mentions (ie, words beginning with “@’”) were removed. Unigrams and bigrams with term frequency-inverse document frequency normalization were used as features. Other features included whether the tweet contained a reference to a hospital staff member and a reference to themselves or a family member. We selected the top 15,000 features from a classifier that produced the highest *F*_1_ score with the lowest overfitting. The classifier was assessed for overfitting by comparing the difference in the performance on the training and test sets.

### Geolocation of Tweets

This study aims to analyze and compare patient experience sentiments at national and regional levels in the country using the Twitter data. However, Twitter data very rarely contain location information. Previous studies have found that a very small fraction of users share their geo-coordinates in the tweets [23]. We also found that only 2.97% (81,930/2,759,257) of the total relevant patient experience tweets contained user-defined geo-coordinates. Therefore, we developed a location inference engine to approximately identify geographical locations, such as country, state, and region of the relevant tweets in this dataset.

We used a combination of information from the users’ profile and GPS (Global Positioning System) coordinates of tweets, when available, to infer the location of the tweets. We also used the Google Maps Geocoding application programming interface [24] in conjunction with the US Census Bureau state boundaries [25] to infer the US state of each tweet. Because a user can input any free text containing a combination of words, symbols, and emojis as location in their profile, we built a library of highly used junk locations (eg, “in your heart,” “with aliens,” “under your bed,” etc) combined with natural language processing (NLP) to identify useless location strings. A list of example location strings is shown in Multimedia Appendix 3. Our geolocation engine was built specifically for social media, wherein users are free to provide any string as their location. It augments Google’s geocoding service [26] with NLP and data mining. This engine performs a list of NLP operations to get rid of irrelevant locations and to parse and format location strings followed by querying to Google Map application programming interface for geolocating the location. We chose to use Google’s geocoding service because it has been repeatedly reported to have a better accuracy [27], thorough coverage [28], and is equipped to handle ambiguous locations [26].

**Table 1:** Example tweets for the patient experience dataset curation.

Tweet class	Examples
*Patient experience*^a^; hand labeled: 3708	After having a tumor removed from my bladder I returned to the ward with a catheter fitted. #cityhospital
*Irrelevant*^b^; hand labeled: 9810	Need tips for better communication with your doctor? #medicine #wellness

^a^Patient experience tweets are defined as tweets related to an individual’s experience in a health care setting.

^b^Irrelevant tweets are any tweets captured in the database that are not patient experience tweets.

There are other geocoding services, such as Nominatim and Carmen, which could have been used in this study. However, there is a limitation to using Nominatim because geolocation is tightly coupled to specific address formats [29], which would be difficult to use with Twitter data because users can specify their location in any format using a free-text field. Additionally, Carmen provides maximum resolution only at the city level for both geocoding and reverse geocoding, which may lead to incorrect results for the users who provide finer-grained locations such as neighborhoods [30]. The location database and alias list of Carmen also needs improvement. The creators of Carmen recommend augmenting the location database and alias list by querying to other search engines and public resources [30]. For this reason, we found that the geolocation engine we built is better suited for the purposes of this study.

Using the geolocation engine, we determined the state location for each tweet and the associated broader region that each state was assigned to. The regions examined in this study were chosen and aggregated as defined by the US Census Bureau, which are each a grouping of states and identified with a single-digit census code [31]. The US Census Bureau groups each region by similarities in historical development, population, and economy and recommends using this framework for comparative efforts [32]. Previous research has shown regional differences in health care [33], and this study sought to determine if regional differences in care could be identified on Twitter. Further details of the tweet extraction, curation, and geocoding are provided in Multimedia Appendix 4.

### Tweet Sentiment

A prime objective of this study is to gauge and compare the sentiments of patient experiences across the country. To compute the sentiments expressed in the tweets, we adopted a widely accepted and used lexicon and rule-based sentiment classifier called Valence Aware Dictionary for Sentiment Reasoning (VADER) [34]. However, we appended VADER’s dictionary and rules to provide a broader representation of Twitter data, which included incorporating more than 110 emojis and their respective sentiment scores [35].

VADER computes sentiment and valence for each word level and provides positive, negative, and neutral scores at the sentence level. We used the compound score, which is a unidimensional and normalized measure of sentiment. It is computed by summing the valence scores of each word in the lexicon, adjusted according to the rules, and normalized to be between −1 (most extreme negative) and +1 (most extreme positive). We used VADER to compute the compound sentiment score for every sentence in the tweet, and then took the mean of all nonzero compound scores to provide a sentiment score per tweet. We considered a sentiment positive if the mean compound score was ≥0.3 or negative if the score was ≤−0.3. Mean compound scores between −0.3 and 0.3 were considered neutral. In the majority of our analyses for this study, we considered tweets with positive and negative scores only because these sentiments provide more actionable data.

### Population Size Examination

We further explored if the patterns of discussion and reporting about patient experience vary by geographical region and by population size of the location of the Twitter users. To perform this analysis, we aggregated the labeled Twitter data with identified state locations into 4 US regions and also dichotomized the data into metropolitan (population ≥50,000) and nonmetropolitan (population <50,000) areas [6].

We used the recent and most detailed geographic polygon data on urban areas from the US Census Bureau [36] to infer if a tweet was from a metropolitan or nonmetropolitan area. According to these data, there are more than 486 urbanized areas (population ≥50,000) and 3087 urban clusters (5,000 ≤population<50,000) in the United States, accounting for a total of 24,356 geographic polygons. The geo-coordinate of each tweet inferred by our location identification engine was checked against these polygons. A tweet was considered metropolitan if the geo-coordinate of the tweet fell inside a geographic polygon of an urbanized area. The tweets falling either inside a polygon of the urban clusters or falling outside all of the urban polygons were considered nonmetropolitan tweets.

### Temporal Examination

The time at which a tweet is posted can be an informative dimension to analyze the patient experience. Certain sentiment patterns, for example, might be more popular during the day than at night. To uncover such patterns, we analyzed Twitter data regarding patient experience by examining the time of the day and that of the week when the tweets were posted. This gives us a broad set of trends to analyze the activity of a selected geographic region.

Because the timestamps of Twitter data are provided in coordinated universal time (also known as Greenwich Mean Time), this analysis requires converting the time at which a tweet was posted onto a Twitter user’s local time. We used the inferred state information provided by sour geolocation classifier along with the time zone information for each state to identify the correct coordinated universal time offset to calculate the local time.

### Statistical Analysis

To determine if there were any differences between the discussions of patient experience on Twitter in metropolitan and nonmetropolitan areas, we performed a Mann-Whitney nonparametric test on the sentiment scores of the tweets. We tested the ranked distribution of metropolitan and nonmetropolitan sentiment scores to determine if they were approximately equal at national and regional levels, aggregating positive and negative scores together. We also compared the metropolitan and nonmetropolitan sentiment scores at national and regional levels by the sentiment polarity and valence. The nonparametric tests were chosen because the sentiment score distribution was found to be symmetric and bimodal.

## Results

### Geolocation of Tweets

After evaluating a set of classifiers, we selected a support vector machine classifier that produced the highest *F*_1_ score with the lowest overfitting. The selected classifier achieved an accuracy of 83% with a precision and recall of 70% and 69%, respectively, for the patient experience tweet class. We filtered the gathered tweets with no URLs and ran the selected classifier to identify patient experience tweets. There were 33.88% of the total tweets (9,252,004/27,309,724) found to be without a URL, out of which 29.82% (2,759,257/9,252,004) were labeled as patient experience by the classifier. We also verified the classifier-labeled patient experience tweets by manually curating a random set of 5000 tweets and found it to be 76% in agreement with the classifier.

To perform national and regional analyses, the labeled patient experience tweets were required to be geocoded. We found that only 2.97% (81,930/2,759,257) of the total patient experience tweets contained geo-coordinates shared by the users. After using our geolocation inference engine, we identified 31.76% (876,384/2,759,257) patient experience tweets that belonged to 1 of the 50 US states, District of Columbia, Puerto Rico, or the United States Virgin Islands; 19.25% (531,062/2,759,257) of the patient experience tweets were from outside the United States, whereas 14.58% (402,295/2,759,257) had insufficient information and 35.14% (969,614/2,759,257) had no information to infer geolocation. Manual curation of 10,000 randomly selected tweets using MTurk validated that 91% (9100/10,000) of the inferred locations through the geolocation engine were correct (with 87%, 8,700/10,000 agreement between 2 MTurk curators). We also verified the quality of the MTurk curators for this task using an in-house team to manually curate the first 2000 tweet geolocations. Our curators had 79% agreement with the MTurk curators.

The further dichotomization of the patient experience Twitter dataset into metropolitan and nonmetropolitan tweets identified 69.36% (607,891/876,384) of total tweets as metropolitan tweets and 30.64% (268,493/876,384) as nonmetropolitan tweets across the 4-year study period from February 2013 to February 2017. The state of Rhode Island was identified as the state with most tweets in a metropolitan area per 100,000 residents (at 97.2%) in the patient experience dataset, and Wyoming had the most tweets in a nonmetropolitan area per 100,000 residents in the patient experience dataset (at 89.7%); 100% of the tweets from the District of Columbia were metropolitan because it is entirely urbanized.

### Tweet Sentiment

Of the 27,309,724 tweets collected between February 2013 and February 2017 using a set of patient experience-related keywords, the classifier was able to identify 2,759,257 tweets that were labeled as patient experience. After running the patient experience tweets through the geolocation classifier, we identified 876,384 tweets by approximate location to use for spatial analyses. At the national level, we observed 27.83% positive (243,903/876,384), 36.22% neutral (317,445/876,384), and 35.95% negative (315,036/876,384) patient experience tweets in the dataset. For this study, we chose to exclude tweets with neutral sentiment scores.

**Figure 1:**
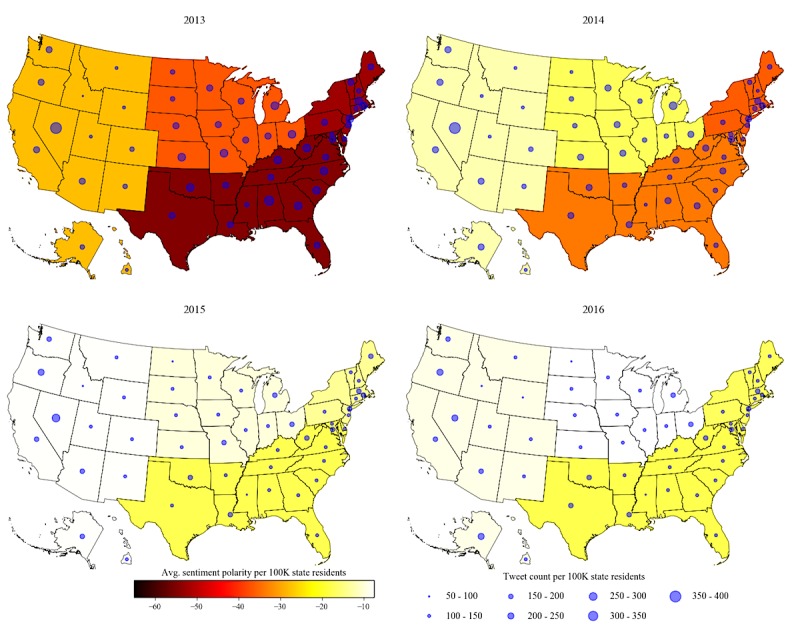
Patient experience tweet sentiment by region over time. K represents thousand, where any number is followed by three zeros (eg, 100K equals 100,000).

**Figure 2:**
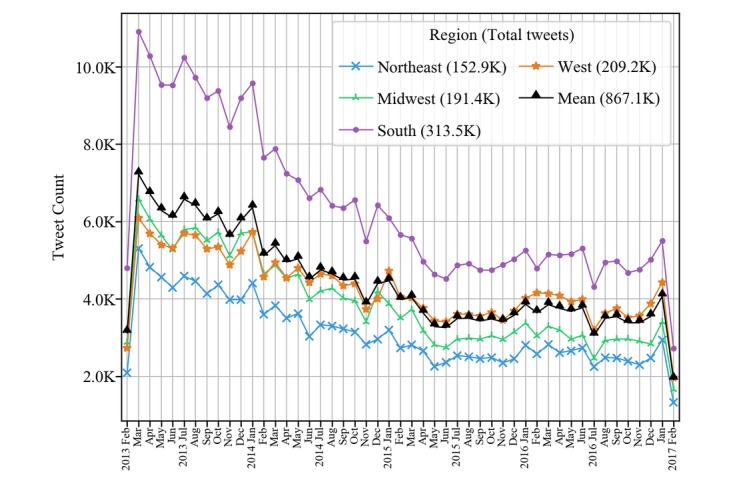
Patient experience tweet volume by region over time. K represents thousand, where any number is followed by three zeros (eg, 100K equals 100,000).

Figure 1 and Figure 2 show the patient experience tweet count and sentiment trends over the 4-year study period across the 4 regions of the United States. The color scale of the 4 regions in Figure 1 represents the average sentiment polarity rate and the blue dot in each state depicts the approximate size of the patient experience tweet rate.

The average sentiment polarity rate is the mean difference in the counts of positive and negative tweets per 100,000 residents in the state; for example, in 2013, there was 54 more negative patient experience tweets for every 100,000 Twitter users in the south region. Likewise, there were 28 more negative tweets in the west region compared with the positive tweets. The patient experience tweet rate is the number of patient experience tweets per 100,000 residents in the state [6]; for example, there were 372 patient experience tweets posted in Nevada, 239 in Texas, and 225 in California in 2013 per 100,000 residents.

Overall, the average sentiment polarity shifted to be less negative every year across all the regions in the United States, as shown in Figure 1. The average sentiment polarity rate for the northeast, midwest, south, and west regions shifted from −52, −37, −54, and −27 in 2013 to −36, −17, −33, and −12, respectively, in 2014. The sentiment polarity further shifted toward less negative scores from 2015 to 2016 in all the regions except for the northeast region, which recorded a sentiment polarity rate of −14 in 2015 compared with −17 in 2016.

Similarly, the patient experience tweet rate also decreased across all the states over the 4-year study period. The number of states with at least 200 tweets per 100,000 residents was reduced from 35 states in 2013 to 3 states (Nevada, Oregon, and Alaska) in 2016. The count of patient experience tweets from February 2013 to February 2017 (a total of 49 months) by region is shown in Figure 2. Overall, the south region posted the highest volume of tweets and the northeast posted the lowest volume of tweets during the study period with a visible downward trend across the 4 regions of the United States.

**Figure 3:**
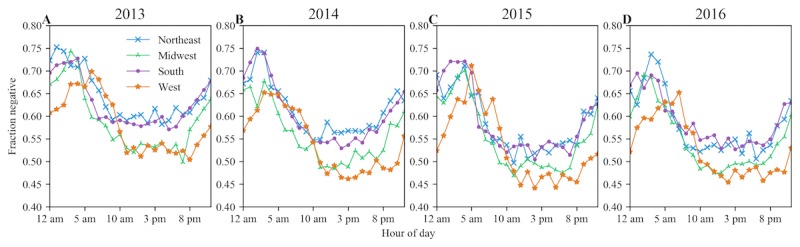
Fraction of negative patient experience tweets by the hour of the day in each region for years 2013-2016.

**Figure 4:**
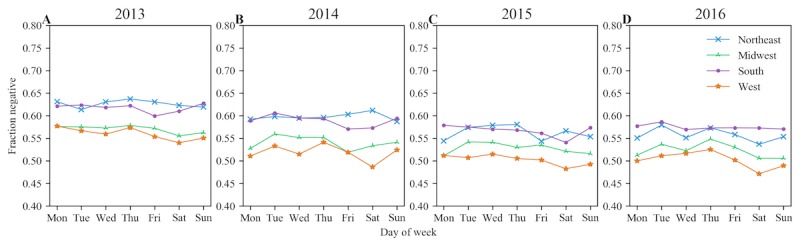
Fraction of negative patient experience tweets by the day of the week in each region for years 2013-2016.

We further examined the negative patient experience tweets with respect to the hour of the day when they were posted. We focused on negative tweets because the average sentiment polarity across all the regions was consistently found to be negative, as shown in Figure 1. Figures 3 and 4 present a set of plots showing the hourly trend and the day-of-week trend respectively for the fraction of the negative patient experience tweets by region for each study year.

The hour-of-day trend revealed that the overall negative tweet fraction exceeded the positive at almost every hour-of-day in all the regions. However, the negative tweet fraction was at its minimum during working hours (8 am-5 pm). The northeast and south regions exhibited very similar tweet patterns during the working hours regardless of the large differences in the tweet counts (Figure 3). The midwest and west regions also show similar patterns to each other. There were similar or higher volumes of positive tweets posted between 10 am and 8 pm in the midwest and west from 2014 to 2016. The fraction of negative tweets was consistently above 0.5 between 10 am and 8 pm in the northeast and south regions.

The day-of-week trend revealed that the overall fraction of negative tweets in all 4 regions was similar over the 4-year study period (Figure 4). The negative tweet fraction was consistently equal to or above 0.5 for all regions in the United States except in the west region in 2015 and 2016. Additionally, Fridays and Saturdays were found to be the least negative days in the week for tweets in the patient experience dataset across all regions and all study years. There was a visible decrease in the negative fraction from Thursday to Friday and a visible increase from Saturday to Sunday in almost all regions every year.

The plots for the hourly and day-of-week tweet counts are shown in Multimedia Appendix 5. We found that the highest number of patient experience tweets was sent from 10 am to 10 pm and on Monday through Thursday across all regions. The south consistently recorded the highest volume of tweets, and the northeast recorded the lowest tweets hourly between 10 am and 10 pm as well as every day of the week. The regional patterns in hourly and day-of-week tweet counts remained similar over the 4-year study period with a visible decrease from 2013 to 2015. Both tweet count trends remained similar across all regions in the years 2015 and 2016.

### Population Size Examination

Using the geolocation classifier, we were able to identify whether a tweet was from a metropolitan (≥50,000 persons) area or a nonmetropolitan (<50,000 persons) area. At the national level, we identified 267,894/867,149 tweets in nonmetropolitan areas, accounting for 30.89% of tweets in the geocoded dataset. We identified 599,255/867,149 tweets in metropolitan areas, accounting for 69.11% of tweets in the geocoded dataset. We excluded the tweets from District of Columbia and Puerto Rico for this examination.

Using the sentiment classifier, we observed at the national level that patient experience-related tweets from nonmetropolitan areas had higher negative sentiment when compared with patient experience tweets from metropolitan areas; however, the difference was small (57.3% vs 55.9%). Similarly, we observed patient experience tweets from nonmetropolitan areas to have a slightly lower percentage of positive tweets compared with those tweets from metropolitan areas (42.7% vs 44.1%).

Regionally dividing the metropolitan and nonmetropolitan tweets revealed that the northeast has the largest fraction of metropolitan tweets i.e., 81.12% (124,135/152,944), followed by the west at 73.28% (153,336/209,246), south at 64.65% (202,710/313,543), and midwest at 62.21% (119,074/191,416). The sentiment comparison across all regions and metropolitan or nonmetropolitan areas found that metropolitan patient experience tweets in the western region were most positive (48.3%), and the nonmetropolitan tweets in the south were most negative (60.1%). However, the sentiment percentage difference between the metropolitan and nonmetropolitan tweets within respective regions was also small. The west held the largest difference in sentiment percentage difference with 51.7% negative tweets in metropolitan areas compared with 53.8% in nonmetros. The northeast recorded the smallest sentiment difference (59.2% negative tweets in metropolitan vs 58.4% in nonmetropolitan).

We further divided the metropolitan and nonmetropolitan tweets to study the yearly patterns within and across the regions. In each study year, we found that more negative tweets were posted than positive in all metropolitan and nonmetropolitan areas across all regions. Tweets in the northeast metropolitan area posted the highest percentage of negative tweets (63.0%) across all the regions in 2013. From 2014 to 2016, the southern nonmetropolitan area consistently had the highest percentage of negative tweets with 60.6%, 57.5%, and 58.2% negative tweets for each of these respective study years, 2014, 2015, and 2016, respectively, in the study. However, the western metropolitan and midwestern metropolitan areas recorded the highest and second highest percentage of positive tweets, respectively, each year in the study. The highest positive tweet percentage of the western metropolitan area was 50.5% and the midwestern metropolitan was 49.2%, and both were recorded in 2016. The difference in sentiment percentages within all regions over the 4-year study period was small. The west reported the largest percentage difference in negative sentiments between metropolitan and nonmetropolitan in 2016, 49.5% vs 52.5%, respectively.

### Statistical Analysis

**Figure 5:**
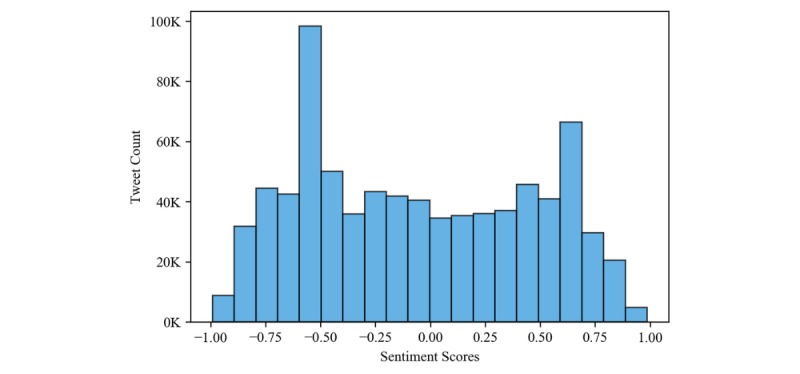
Sentiment score distribution of all tweets (n=788,904, µ=−0.06, and SD 0.509).

In further investigations, we performed statistical tests to identify if there were any significant differences between the sentiment scores of the metropolitan and nonmetropolitan tweets. The shape of score distribution was found to be symmetric bimodal with local maxima on either side of the origin, as seen in Figure 5. Hence, we performed the Mann-Whitney nonparametric test to check if the ranked distribution of the sentiment scores from the metropolitan and the nonmetropolitan areas were approximately equal.

We performed the statistical tests on the sentiment scores at both national and regional level. The sentiment score data were also divided into the following 4 quantiles: Q-1 (0.0, 0.25), Q-2 (0.25, 0.5), Q-3 (0.50, 0.75), and Q-4 (0.75, 1.0) for the analysis. These quantiles represent the relative polarity of the data; for example, the tweets in Q-1 can be viewed as extremely negative compared with the extremely positive tweets in Q-4. Similarly, the tweets in Q-2 and Q-3 can be viewed as mildly negative and mildly positive within a dataset. The descriptive statistics and *P* values of all the statistical tests are shown in Table 2. The table also shows the Cohen *d* effect size for the tests that found significant differences.

The sentiment scores of the metropolitan tweets at the national level were found to be significantly different to the nonmetropolitan tweets (*P*<.001). The sentiment scores of the midwest, south, and west regions’ metropolitan tweets were also found to be significantly different from the nonmetropolitan tweets at alpha=0.1%. The *P* value for the northeast region was .003.

After dividing the data into quantiles, the analysis established that the statistical significance could vary at different quantiles and that it was irrespective of the results that we found for the data without dividing it. Nationally, the difference between the metropolitan and the nonmetropolitan tweets was found to be statistically significant for data quantiles Q-1 and Q-3 (*P*<.001). 

**Table 2:** National and regional descriptive statistics and nonparametric test results of patient experience tweet sentiments in metropolitan and nonmetropolitan areas.

Tweet region and sentiment score quantiles^a^		Metropolitan tweets		Nonmetropolitan tweets		*P* value	Cohen *d* effect size^b^
		n (%)	Mean (SD)	n (%)	Mean (SD)		
	All tweets	544,962 (69.08)	−0.056 (0.511)	243,942 (30.92)	−0.068 (0.505)	<.001	0.023
	Q-1	152,796 (68.87)	−0.698 (0.127)	69,067 (31.13)	−0.655 (0.126)	<.001	0.341
	Q-2	150,712 (68.36)	−0.351 (0.135)	69,768 (31.64)	−0.349 (0.136)	.002	N/A^c^
	Q-3	137,361 (68.94)	0.151 (0.156)	61,876 (31.06)	0.148 (0.156)	<.001	0.019
	Q-4	138,339 (70.14)	0.621 (0.128)	58,905 (29.86)	0.620 (0.128)	.04	N/A
**Northeast**							
	All tweets	112,242 (81.08)	−0.086 (0.508)	26,184 (18.92)	0.078 (0.503)	.003	N/A
	Q-1	33,560 (81.66)	−0.660 (0.127)	7536 (18.34)	−0.659 (0.127)	.30	N/A
	Q-2	32,323 (81.01)	−0.354 (0.134)	7575 (18.99)	−0.345 (0.136)	<.001	0.066
	Q-3	27,349 (80.39)	0.150 (0.159)	6671 (19.61)	0.152 (0.160)	.17	N/A
	Q-4	26,321 (81.29)	0.619 (0.131)	6059 (18.71)	0.619 (0.128)	.42	N/A
**Midwest**							
	All tweets	108,453 (62.15)	−0.035 (0.512)	66,043 (37.85)	−0.051 (0.508)	<.001	0.032
	Q-1	29,045 (61.70)	−0.657 (0.126	18,027 (38.30)	−0.656 (0.126)	.27	N/A
	Q-2	29,085 (61.18)	−0.350 (0.136	18,454 (38.82)	−0.347 (0.136)	.12	N/A
	Q-3	27,877 (62.44)	0.153 (0.155)	16,770 (37.56)	0.149 (0.156)	.002	N/A
	Q-4	28,957 (63.26)	0.623 (0.126)	16,814 (36.74)	0.621 (0.127)	.08	N/A
**South**							
	All tweets	183,829 (64.67)	0.080 (0.506)	100,448 (35.33)	−0.092 (0.50)	<.001	0.024
	Q-1	54,537 (64.39)	−0.656 (0.127)	30,163 (35.61)	−0.652 (0.126)	<.001	0.032
	Q-2	52,931 (63.93)	−0.353 (0.136)	29,859 (36.07)	−0.353 (0.136)	.27	N/A
	Q-3	45,676 (64.39)	0.149 (0.158)	25,263 (35.61)	0.147 (0.158)	.04	N/A
	Q-4	43,424 (66.05)	0.617 (0.129)	22,324 (33.95)	0.616 (0.128)	.20	N/A
**West**							
	All tweets	140,438 (73.26)	−0.015 (0.514)	51,267 (26.74)	−0.039 (0.511)	<.001	0.048
	Q-1	35,654 (72.77)	−0.660 (0.128)	13,341 (27.23)	−0.659 (0.126)	.44	N/A
	Q-2	36,373 (72.38)	−0.345 (0.134)	13,880 (27.62)	−0.346 (0.134)	.02	N/A
	Q-3	36,459 (73.46)	0.153 (0.152)	13,172 (26.54)	0.149 (0.154)	.002	N/A
	Q-4	39,637 (74.30)	0.626 (0.126)	13,708 (25.70)	0.625 (0.127)	.31	N/A

^a^The 4 sentiment score quantiles are shown using Q-1 (0.0, 0.25), Q-2 (0.25, 0.5), Q-3 (0.50, 0.75), and Q-4 (0.75, 1.0). The results are reported at α=0.1%.

^b^The Cohen *d* effect size was computed for tests that found significant differences.

^c^N/A: not applicable.

This result implies that the extremely negative and mildly positive subset of the metropolitan tweets was significantly different than their counterpart tweets from nonmetropolitan areas at the national level. At the regional level, we found statistically significant differences only for Q-2 in the northeast and Q-1 in the south region. The effect size analysis showed that the metropolitan and nonmetropolitan tweets with extremely negative sentiments (ie, Q-1) had a medium effect size (*d*=0.341) at the national level. The remaining tests showed a low side effect.

## Discussion

### Principal Findings

Our findings suggest that Twitter is a unique platform for identifying differences in health care and sentiment of discussion across various geographical perspectives over the 4-year study period. The methodologies developed in this study present an informative examination of the sentiments of patient discussions of health care online. By identifying the opinions and attitudes of patients using social media, we can supplement traditional measures of collecting feedback to better understand the care received across the United States. This study has developed or built upon methodologies to examine social data from various geographical perspectives, including national, regional, and population levels across a 4-year study period.

We found that tweets related to patient experience lean toward a higher percent negative sentiment at the national level. Previous research suggests that patient experience scores are directly related to specific factors of care, such as wait time, the quantity of nurses or doctors at the health care facility, or even cost of care [3,4]. Hospital care in the United States has been found to be generally positive [37], and polling measures have found that Americans generally rate their health care experience as good [38,39]. However, this study is not restricted exclusively to hospital data and encompasses a larger scope of care outside of hospitals, which may be attributed to this discrepancy. Web-based examinations of patient experience may differ from what is being reported in interview- and survey-based reports of care. This study also found slight differences in patient experience tweet sentiments that varied owing to region and population. We observed higher percent positive tweets related to patient experiences in the northeast region as well as areas that are defined as metropolitan with a population of ≥50,000 residents. This supports research on the geographic variability of health care cost and outcomes, which can be reflected through Web-based sentiment scores [33,40]. Further research based on these observations can provide insight into the type of care provided in these areas. The sentiment of patient experience tweets in this study over the 4-year study period gradually skews to less negative, which supports previous reports that found that hospital patient experience trends demonstrate positive progress in multi-year evaluations [41].

We observed a downward trend in the tweet volume during the 4-year study period, whereas tweet sentiment was found to increase across all 4 regions of the United States. This trend could be attributed to either a decrease in percent negative tweets posted over time or an increase in percent positive tweets over time for patient experience discussions. Additionally, although Twitter has not publicly commented on this, researchers and developers who work with this platform have observed a decline in Twitter usage in the United States since 2014[42]. This observation may explain why this study also experienced a constant decrease in patient experience tweet count over the 4 study years.

This study provides an in-depth presentation of the time of the day a tweet was posted. We observed the sentiment of tweets to have a lower negative fraction during daytime hours, whereas the sentiment of tweets posted between 8 pm and 10 am tended to have a higher negative fraction. This observation was seen across all 4 regions of the United States. This observation supports previous research that shows that patient care can be compromised during night hours and on weekends particularly because this is a time when facilities may be closed or have reduced staffing [43]; for example, lower survival rates for postcardiac arrest patients have been observed during night and weekend care [44], and measures to improve safety during off-hours care have been recommended [45]. Further research into the significance of these observations is needed.

By examining the differences in tweet sentiment between metropolitan and nonmetropolitan areas, we sought to determine if the discussion of care online differs based on population size. We found that metropolitan areas across the United States have higher percent positive tweets compared with nonmetropolitan areas, which supports research on differences in health care in rural populations compared with the care in urban populations [7]. Metropolitan cities have been found to have better access to care because many have large health care institutions and resources nearby that smaller communities lack [7,8]. Although there are some noted disadvantages to access to care in more populated cities, including longer wait times, travel times, and appointment availability, we would have expected the sentiment of tweets between the metropolitan and nonmetropolitan areas to have a larger difference, which was not observed in this study. Although we do observe statistically significant results in the associations between certain sentiment quantiles and population size based on the metropolitan and nonmetropolitan areas, we do recognize that this is a large-scale dataset and the impact of the results are weak at best. Further research could provide better insight into care expectations and the Web-based conversations between varying population levels in the United States.

### Limitations

There were several limitations to our study. First, selection bias could occur from the nature of Twitter usage, a platform which is heavily comprised of adults aged 18 to 29 [46]. Representation of tweets may not be evenly distributed across all age groups. Second, we collected our data based on selected keywords related to patient experience, which may not have captured all tweets on the subject matter. Owing to the broad nature of the intended dataset, there is a chance some discussions of patient care were missed. There are limitations to the selected classifier for identifying patient experience tweets as well. We found that the selected classifier achieved an accuracy of 83% with the precision and recall of 70% and 69%, respectively, for the patient experience tweet class, which suggests there is still a chance that tweets discuss health care, but perhaps a tweet that is not an exposure to health care could be captured in this dataset. As previously noted, we observed a decrease in the tweet volume over the 4-study year period, which could indicate that people are posting less on Twitter over time. To minimize the bias of the tweet count, we normalized the patient experience data using yearly state population estimates or yearly tweet counts in our analysis. We present the count data as supplemental information in the analysis. The effect of decreasing tweet counts may introduce bias in the observed data, and this needs to be explored further.

Additionally, there are limitations to state identification because the human validation of our geocoding engine has 91% accuracy. Therefore, there is a 9% chance of error in the inferred states. The errors in the geolocation are primarily owing to the way users provide their location information in their profile; for example, if a Twitter user provides only a city name with no state or country information included in the location field in her profile, the inferred state might be incorrect. Furthermore, querying any location in our engine produces a list of possible options for the state and country. However, we can only select 1 out of all potential options. We are currently choosing the first one on the list.

Finally, our location engine, which infers the state and if a tweet is from a metropolitan or nonmetropolitan area, is based on boundary polygon data on state and urban areas provided by the US Census Bureau. Although we used the highest available data resolution, the inferred location might be incorrect if the tweet geo-coordinates fall very close to the polygon boundaries. There is also a limitation of using a denominator of “per 100,000 residents per state” for our understanding of this dataset. We acknowledge that this denominator represents neither patients on Twitter per state nor Twitter users per state. We used this denominator as referenced by state population US Census Bureau data.

There are ethical considerations that must be considered when using data from social media sites such as Twitter. Understandably, users have concerns about privacy and confidentiality of information posted online. Interestingly, Web-based data used for social benefit or public health interest are often perceived to be more acceptable in social media research among users [47,48]. Even though we acknowledge the concerns of users, all information used in this study was acquired for academic research and was restricted to publicly available posts that users have selected not to post privately. Additionally, we attempted to address concerns about privacy and confidentiality by analyzing and disseminating aggregated numerical data only.

### Future Direction

The findings of this study have implications for future research examining patient feedback online and the usefulness of the knowledge it can provide. Twitter can be prospectively or historically monitored by geographical location to determine how patients feel about the care they receive. This novel approach presents patients with the opportunity to freely discuss their feedback on all aspects of care provided without being limited to the restrictions from more traditional structured questionnaires. Although a user-based approach was outside the scope of this study, future research using the methodologies presented could consider analyzing user-specific data to further examine geographical and temporal differences in patient experience discussions. Additionally, Twitter surveillance of Web-based discussions may provide health care providers, health institutions, and policy makers with both positive and negative trends in the care received in their jurisdiction. This can inform stakeholders of where health care can be improved, particularly during a time when the influence of patient engagement can direct where limited resources should be allocated. Furthermore, these data have the power to provide future research into differences of patient feedback between population demographics, topics of discussion, or even questions to understand if patients are receiving the right care at the right time. Deeper knowledge on the discussions of care online can provide valuable and insightful information, which has the power to influence how health care is provided across the United States.

### Conclusions

This study presents methodologies for a deeper understanding of Web-based discussion related to patient experience across space and time. Twitter, as a social media platform, provides a unique and unsolicited perspective from users. This characterization of data provides a unique opportunity to examine geographic and temporal differences in the sentiments of patient opinions and feedback. The findings provided in this study can lead to further research and understanding of the culture of health in the United States as provided by real-time social data.

## Appendix

Multimedia Appendix 1 Keyword classes, keywords, and example rules that were used to extract the patient experience Twitter data.

Multimedia Appendix 2 Amazon mechanical turk curation guide.

Multimedia Appendix 3 Tweet classification.

Multimedia Appendix 4 Tweet extraction, curation, and geolocation flowchart.

Multimedia Appendix 5 Patient experience tweet counts by the hour-of-day and day-of-week for each US region from years 2013 to 2016.

## Abbreviations

GPS: Global Positioning System
MTurk: Amazon Mechanical Turk
NLP: natural language processing
VADER: Valence Aware Dictionary for Sentiment Reasoning
