# Supplementary material for: Using Twitter to Examine Web-Based Patient Experience Sentiments in the United States: Longitudinal Study
Source: J Med Internet Res. 2018 Oct 12;20(10):e10043. doi: 10.2196/10043 (PMC6231860; doi:10.2196/10043)
Supplement: Multimedia Appendix 1 [file jmir_v20i10e10043_app1.pdf]

Appendix I

The patient experience-related Twitter data was extracted using a paid GNIP license. We used a set of keyword classes and rules to obtain the Twitter data for the study. Keywords were divided into the classes to correctly form the rules. A list of classes along with the corresponding set of keywords, and example rules are shown in the Table S1.1. Note that keyword classes represent different subject of patient experience but may contain common keywords.

A rule may include multiple keyword classes appended using logical operators. For example, a rule for extracting patient experience data related to hospital staff providing hospital care would include keywords from *Hospital Staff* and *Care Condition* classes. Similarly, a rule to extract hospital bills and insurance related patient experience data would look like '*Hospital Bills* AND (*Medical Facility* OR *Hospital Staff* OR *Medicine*)'. We used total fifteen rules to obtain the Twitter data.

Table S1.1. Keyword classes, keywords, and example rules that were used to extract the patient experience Twitter data.

**Keyword Classes**	
Class	Keywords
Medical Facility	Hospital, clinic, urgent care, emergency room, ED
Hospital Staff	Nurse, doctor, medical professional, registered nurse, dr, patient rep
Treatment	Treatment, treat, assist, care
Common Procedures	Surgery, IV, blood
Emergency Care	ICU, NICU, urgent care, emergency room, triage, ED
Hospital Bills	bill, doctor bill, health insurance
Care Condition	Monitor, heal, recover, care, cure, dying, dead, sicker, sick, ill, illness, condition
Hospital Visit	Hospital stay, hospital visit, emergency room visit
Pain	Pain
Medicine	Medicine
Patient Experience	Hospital experience, patient experience
Other	Health, patient, trauma, surgery, critical, procedure, symptoms

Example Rules

1. *Medical Facility* AND *Hospital Staff*
2. *Hospital Staff* AND *Care Condition*
3. *Hospital Visit* AND *Hospital Staff*
4. *Hospital Bills* AND (*Medical Facility* OR *Hospital Staff* OR *Medicine*)
5. *Medical Facility* AND *Common Procedures*
