# Supplementary material for: Using Twitter to Examine Web-Based Patient Experience Sentiments in the United States: Longitudinal Study
Source: J Med Internet Res. 2018 Oct 12;20(10):e10043. doi: 10.2196/10043 (PMC6231860; doi:10.2196/10043)
Supplement: Multimedia Appendix 2 [file jmir_v20i10e10043_app2.pdf]

Appendix II

We used Amazon Mechanical Turk to crowdsource the curation of the Patient Experience Twitter dataset. All Amazon Mechanical Turk curators were restricted to only curate each tweet once. Detailed instructions given to all Amazon Mechanical Turk Workers are below:

Title: Identifying Patient Experience Tweets
Description: You will determine if a tweet is related to patient experience
Keywords: classification, tweet, twitter, patient experience
Number of assignments per task: 2 (i.e. how many unique workers do you want to work on each task)
Time allotted per assignment: 1 hour
Require that Workers be Masters to do your task: Yes

Instructions:
Does this tweet contain a comment related to patient experience?

Patient experience encompasses all aspects of the individual's (or relative/friend's) healthcare journey, which includes but is not limited to, the individual's decision to receive care, the care experience, or any post-care interactions. Support for a patient is not considered patient experience.

[Note: Usernames and URLs within the tweets have been de-identified]

<u>Example Categorization:</u>

Patient Experience: After having a tumor removed from my bladder I returned to the ward with a catheter fitted. #cityhospital

Irrelevant: Need tips for better communication with your doctor? #medicine #wellness

Task:
Determine if this tweet contains a comment related to patient experience:
Select

- Patient Experience
- Irrelevant
- Unsure
