# Supplementary material for: Using Twitter to Examine Web-Based Patient Experience Sentiments in the United States: Longitudinal Study
Source: J Med Internet Res. 2018 Oct 12;20(10):e10043. doi: 10.2196/10043 (PMC6231860; doi:10.2196/10043)
Supplement: Multimedia Appendix 3 [file jmir_v20i10e10043_app3.pdf]

Appendix III

Table S2.1. Example user location strings that were used by the developed geolocation engine to infer US states for the Patient Experience tweets. The strings are divided into various groups to show the examples of variations in users' input.

**Task: Infer US State for *Patient Experience* tweets**		
**Location Type**	**Example**	**Inferred State**
Simple	Boston	Massachusetts
	Cali babyyyy!	California
Alternative names	The Windy City	Illinois (another name for Chicago)
	Lone Star State	Texas
Multiple places	Chicago/L.A	Illinois
	NYC/LONDON/LA	New York
Less Known	ICDC College	California
	The Land of Oz	North Carolina (a theme park)
Junk	Space with the Aliens	(Useless location string)
	Day Dreaming…	(Useless location string)

Table S2.2. Examples of positive, negative, and neutral Patient Experience tweets.

**Task: Infer Patient Experience Sentiments**	
**Tweet Class**	**Examples**
Positive	Thank God I went to urgent care yesterday
	Surgery went well, in some pain but they have drugs so im ok
Negative	nothing is helping. I'm out of pain medicine :(
	At hospital for about an hour with an emergency but still waiting for the doctor
Neutral	I want to go to the nurse to go home "sick" and go to the park
	I'm 19, why am I having surgery in a children's hospital?
