# Supplementary figures and images for: Using Twitter to Examine Web-Based Patient Experience Sentiments in the United States: Longitudinal Study
Source: J Med Internet Res. 2018 Oct 12;20(10):e10043. doi: 10.2196/10043 (PMC6231860; doi:10.2196/10043)

## Appendix IV:

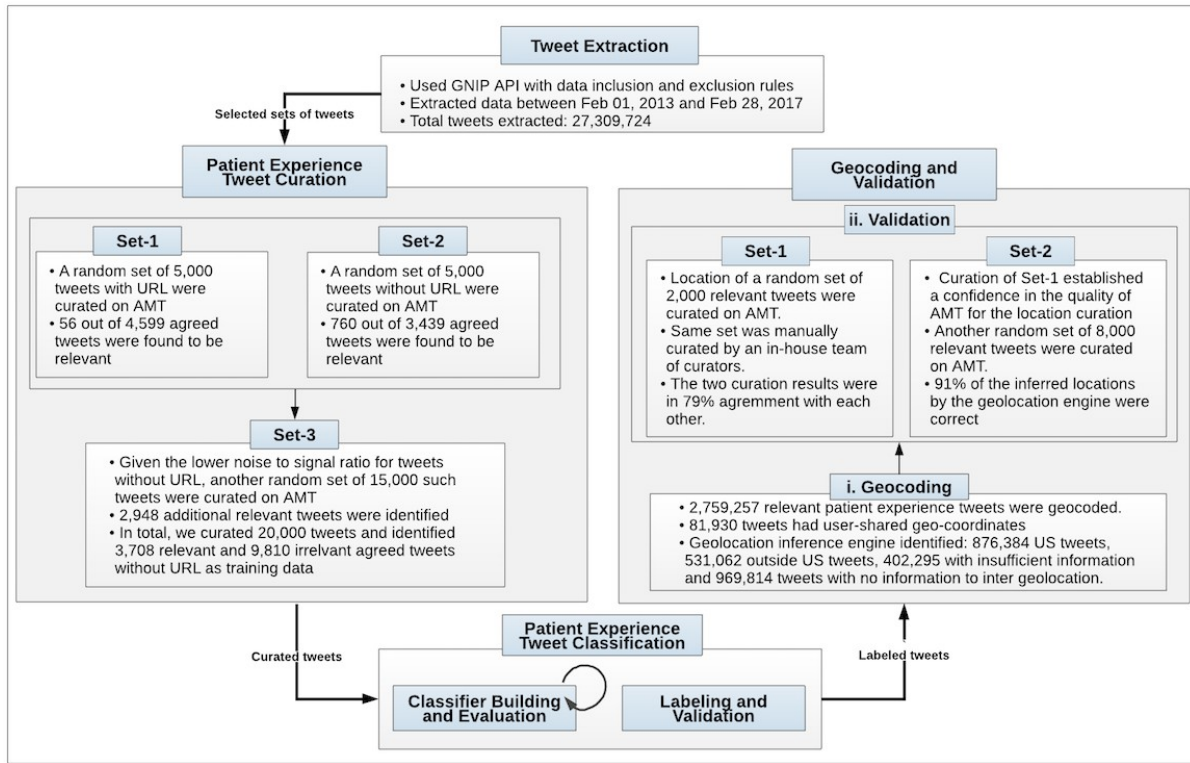

Supplement: Multimedia Appendix 4 [file jmir_v20i10e10043_app4.pdf]
