# Supplementary material for: Using Twitter to Examine Web-Based Patient Experience Sentiments in the United States: Longitudinal Study
Source: J Med Internet Res. 2018 Oct 12;20(10):e10043. doi: 10.2196/10043 (PMC6231860; doi:10.2196/10043)
Supplement: Multimedia Appendix 5 [file jmir_v20i10e10043_app5.pdf]

Appendix V

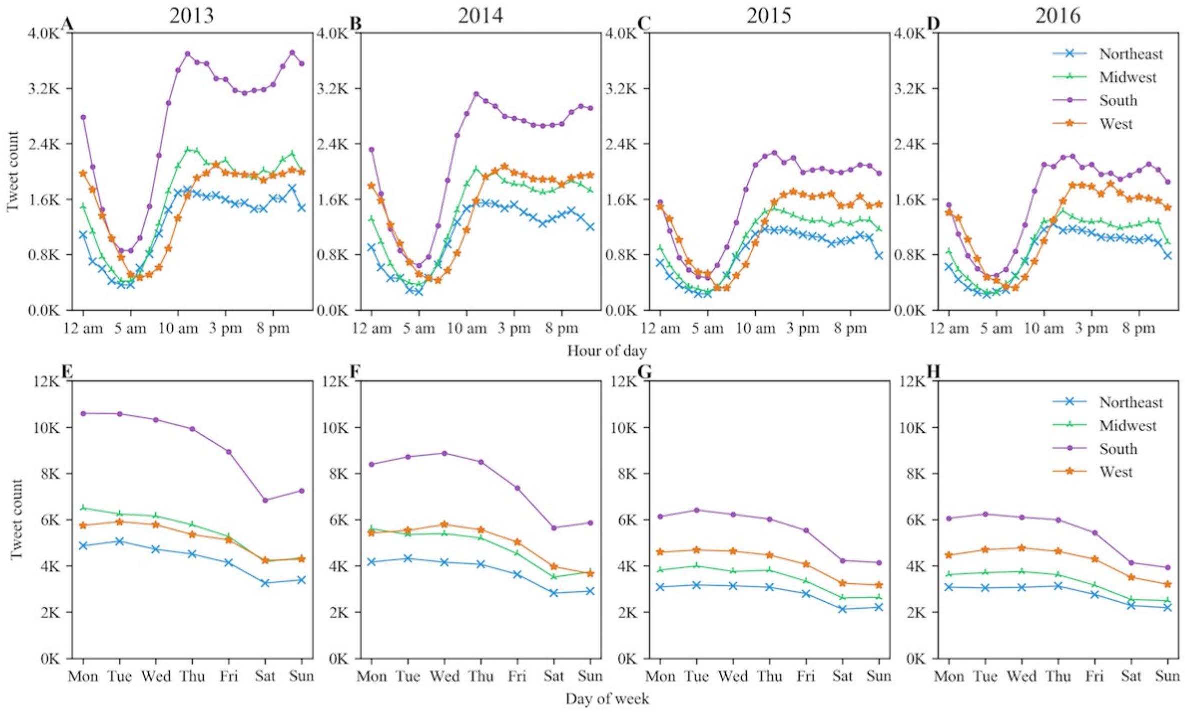


Figure S3.1. *Patient Experience* tweet counts by the hour-of-day and day-of-week for each US region from years 2013 to 2016.
